# Application of Dual Focused Ultrasonic Phased Array Transducer in Two Orthogonal Cross-Sections for Inspection of Multi-Layered Composite Components of the Aircraft Fuselage

**DOI:** 10.3390/ma13071689

**Published:** 2020-04-04

**Authors:** Renaldas Raišutis, Olgirdas Tumšys

**Affiliations:** Ultrasound Research Institute, Kaunas University of Technology, Baršausko str. 59, LT-51368 Kaunas, Lithuania; olgirdas.tumsys@ktu.lt

**Keywords:** ultrasound, dual focusing, phased array, single-side access, convex lens, multi-layered composite, dissimilar materials, GLARE, delamination defect

## Abstract

Our previous studies have shown that the application of the proposed technique of a dual focused ultrasonic beam in two orthogonal cross-sections in passive (elevation) and active (azimuth) apertures of linear ultrasonic phased array transducer (ULPAT) enhances the 3D spatial resolution in the case of the inspection of conventional defects (flat bottom holes) or measurement of thickness of multi-layered metal composites. The objective of this work is to apply the proposed technique to enhance the spatial resolution of the ULPAT in the cases of detection and sizing demonstration of internal defects possessing spatially complex geometry, and during the inspection of defective multi-layered thin composite components (e.g., GLARE) of the aircraft fuselage. The specially prepared aluminium specimen possessing an internal defect of complicated geometry (crescent-shaped) was investigated. The simulation results and experiments demonstrate the resolution enhancement, higher amplitude of the reflections (e.g., 2.5 times or +8 dB) and spatial improvement in the defect detection even in the case of the non-perpendicular incidence of ultrasonic waves to the complex geometry surface of the internal defect. During the experiments, the multi-layered GFRP-metal based composite sample GLARE 3-3/2 was investigated in the case of the single-side access to the surface of the sample. The internal artificial delamination type defect of 25 mm was detected with a higher accuracy. Compared to the limitations of conventional ULPAT, the relative error (32%) (at the −6 dB level) of lateral defect dimensions estimation was completely reduced.

## 1. Introduction

The recent demand for lightweight constructions of aircraft frames enables the creation of a variety of new multi-layered and composite materials that differ in their properties and potential applications. At the same time, the quality control of such complicated materials is extremely important to ensure the safety of construction. Various non-destructive methods are widely used to solve these problems [1,2,3]. The most popular ultrasonic methods provide them with a wide range of options for measuring the parameters of different materials [4,5]. However, not all modern materials with their unique properties can be investigated using ultrasonic methods. The creating and joining of new materials and, at the same time, the development of new, fast and reliable inspection methods are integral processes.

One type of the multi-layered composite materials used as constructional material for the AIRBUS A380 is GLARE [6]. GLARE consists of combination of glass fibre reinforced composite (GFRP) layers and aluminium layers. The specific arrangement of GFRP and metal layers allows for the achievement of the necessary material properties for specific application conditions defined by fatigue, stress, etc. However, the application of non-destructive testing methods to test the structure of such materials during development, manufacturing and/or in-service inspection is complicated enough due to the presence of dissimilar materials (e.g., metal and fibre reinforced composite) layers possessing different material and acoustic properties.

There are different existing types of GLARE construction [7]. The main constructional component is prepreg reinforced by unidirectional glass fibres and FM 94 adhesive. Such prepreg layers are laid-up between aluminium sheets. During the manufacturing of multi-layered structure GLARE, different defects could occur, such as disbonds, delaminations, voids, inclusions, etc. Thee detection and identification of these defects in the GLARE samples is an important task for quality control and safety assurance in the aerospace sector [6,8,9,10]. The pulse-echo setup and ultrasonic multi-element phased array transducers (ULPAT) are used for the inspection of GLARE-type materials using ultrasonic bulk waves [11]. In the simplest case, the ULPAT is positioned parallel to the surface of the object being investigated. Furthermore, electronic linear scanning is performed with a focus on the excited beam at a particular depth of the sample [12]. The non-destructive (NDT) testing of the GLARE sample to detect the prepreg gaps was demonstrated by phased array setup (frequency 5 MHz) with a conventional delay line and C-scan imaging by the through-transmission technique based on the single element transducers (10 MHz) and water-jet configuration [13]. The ULPAT of 64 active piezoelements operating at the frequency of 5 MHz was used. The obtained results show the possibility to characterize the gap within thin laminates [13]. In addition, the investigation and evaluation of internal defects (overlaps and gaps) was performed by phased array testing, C-scan acquisition and attenuation analysis over gap regions [7,13]. The central frequency of single-element transducers in the through-transmission arrangement was 10 MHz. It was stated that this is the most sensitive frequency for assessment of GLARE quality [7,13,14]. The ultrasonic NDT procedure was developed in order to inspect the composite laminates in the presence of overlaps and gaps [7,13,14]. However, it does not cover the detection of delamination defects within the composite panels. The application of phased array testing (frequency 5 MHz) and immersion through-transmission setup has been demonstrated for detection of delamination damages due to impacts [10]. However, the technique is not suitable for in-service applications and requires access to both sides of the specimen [7,8,10].

The objective of this work is to apply the technique we proposed to enhance the 3D spatial resolution of ULPAT in the cases of detection and sizing demonstration of internal defects possessing spatially complex geometry, and during the inspection of defective multi-layered thin composite components (e.g., GLARE) of the aircraft fuselage.

These particular tasks cover the achievement of higher accuracy of defect sizing inside the composite structure of joined dissimilar materials (e.g., lightweight metals and fibre reinforced plastics) and their possibility to be applied in the case of only single-side access to the surface of the sample.

The solution is based on the application of a specially designed delay line with the fluid-based convex cylindrical lens [15], which was proposed for contact type inspection during quality control, NDT and material characterization of the multi-layered composites fabricated from joined dissimilar materials (e.g., metal and GFRP). The obtained results demonstrate that the spatial resolution of the 3D ultrasonic beam of ULPAT and the accuracy of defect sizing might be improved by applying the technique of using a spatially dual focused beam in two orthogonal cross-sections, both by lens and electronically.

## 2. Application and Verification of the Independent Spatial Dual Focusing Technique

The contact type linear ULPAT with delay lines can be used for the fast pulse-echo detection of different type internal defects (e.g., delaminations) located at different spatial depths of the specimen under investigation [6]. However, the varying thickness and number of aluminium sheets and intermediate GFRP prepreg layers of the GLARE structure complicates the inspection. It is extremely difficult to focus the ultrasonic beam of ULPAT in a thin GLARE structure [6,10]. In addition, a low lateral resolution occurs in one plane (elevation). To solve this problem, the adapted method, based on the acoustic lens principle from medical research [12], was developed by us and used to achieve the fixed distance focusing of the 1D array in the elevation plane [15].

**Figure 1:**
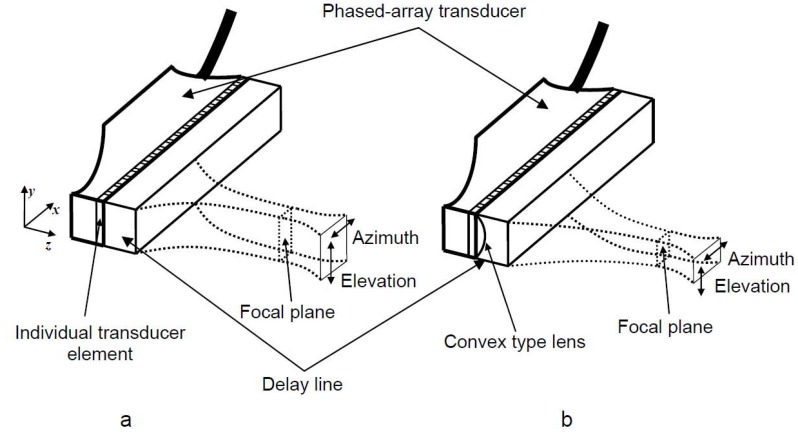
Focusing of the ultrasonic phased array transducer using the different types of the delay line: in azimuth cross-section only (**a**), in azimuth cross-section and in elevation cross-section with convex lens (**b**).

The adapted method presented in our previous study [15] enhances the spatial resolution, applying the dual focusing technique of ULPAT in two orthogonal cross-sections (electronically phasing excitation of elements and geometrical focusing by a lens): active apertures of phased array (azimuth) (Figure 1a) and passive apertures (elevation) (Figure 1b) [15]. The method allows us to avoid the time domain overlapping of the reflected wave signals from the internal structure of the specimen over the signals due to reverberations of each element of the array [15].

**Figure 2:**
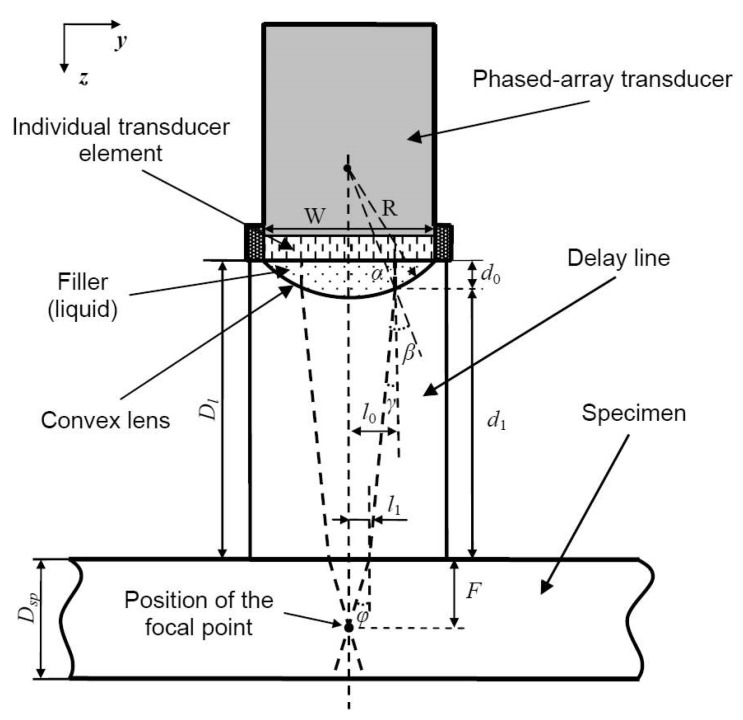
Calculation of the position of the defined point (*F*) of focusing inside the sample (having thickness of *D_sp_*).

In order to realize focusing of the excited ultrasonic beam in the elevation cross-section, the specially designed delay line with a liquid-filled convex type lens (designed and implemented within the housing of such delay line) is suggested to be used in the case of the contact testing procedure of the relatively thin samples possessing internal defects [15]. The convex lens was realized by mechanically milling the cylindrically curved surface within the housing of the delay line and flooding this cavity with acoustic coupling liquid (Figure 2) [15].

The position of the defined point (*F*) of focusing inside the sample with thickness *D_sp_* was calculated by the following equations, taking into account the particular arrangement of the delay line presented in Figure 2 and refraction of ultrasonic rays by Snell’s law:

(1) $$\sin\alpha =\frac{l_{0}}{R}$$

(2) $$\sin\beta =\frac{c_{2}\sin\alpha }{c_{1}}=\frac{c_{2}l_{0}}{c_{1}R}$$

(3) $$F=\frac{l_{1}}{tg\phi }=\frac{l_{0}-\left(D_{l}-d_{0}\right)\cdot \tan\left(\beta -\alpha \right)}{\tan\phi }$$

(4) $$d_{0}=R\cos\alpha -\sqrt{R^{2}-W^{2}/4}$$

(5) $$\phi =\arcsin\left(\frac{c_{3}\sin\left(\beta -\alpha \right)}{c_{2}}\right)$$

where *R* is the radius of cavity cylindrical curvature (the internal shape of the lens), *l*_0_ is the distance in lateral direction between the vertical axis of lens symmetry and the spatial refraction point of particular ultrasonic geometrical ray at boundary of two mediums (the lens filled by liquid and the delay line material), *c*_1_ and *c*_2_ are velocity values of ultrasonic waves in the water-based filler (cavity bellow the active transmitting-receiving surface of the phased-array transducer) and the delay line (material Plexiglas). In addition, *c*_3_ and *φ* are the ultrasound velocities and the refracted angle of bulk ultrasonic waves in the sample under investigation, *D*_l_ is the thickness of the Plexiglas delay line.

The proposed solution of the ULPAT spatial focusing (3D) in the azimuth and elevation cross-sections was tested by using the designed and produced in a lab delay line [15]. The proposed concept was successfully verified by modelling and the appropriate experiments of inspection of specially designed and produced thin metal (steel) sample possessing internal defects, such as artificially made flat-bottom holes (FBH) of different dimensions (diameters and depths) [15]. This technique was also applied for detecting boundaries of the intermediate complicated layer-insert of pressed metal powder (aluminium) within the three-layer specimen of the metallic foam-based composite [15].

**Figure 3:**
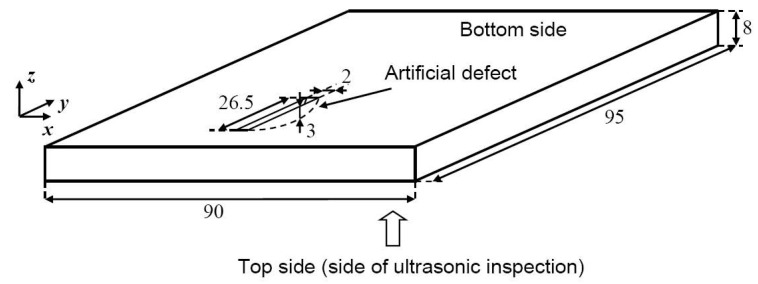
The setup of the investigated aluminium sample possessing artificial crescent-shaped defect (all dimensions are provided in mm, view from the bottom side).

The thin layers of the GLARE structure and significant difference of acoustic impedances between the aluminium and the GFRP layers lead to a non-uniform attenuation characteristic in the frequency range of 5–15 MHz. In this case, it is relevant to investigate the different propagation and refraction trajectories of ultrasonic waves. For this purpose, an aluminium sample with crescent-shaped artificial defect was specially manufactured by a cutting machine (disk-shape cutter) (Figure 3). The spatially complicated geometry and internal surface of the defect was specially selected in order to assess the efficiency of the scanning using the proposed method and to obtain reflections from the surface of such defect in the case of non-perpendicular incidence of ultrasonic waves. This creates challenges for ultrasonic inspections as waves reflected by the curved surface of the defect travel at different propagation and refraction trajectories.

The width of the defect along the *y* axis is 26.5 mm, the width along *x* axis is 2 mm and the maximum height along the *z* axis is 3 mm. Aluminium, as the material for the above-mentioned sample, was selected due to wide use of different aluminium alloys in various metal-based or metal composite-based aviation constructions. In this study, the thicknesses of the manufactured focused (with convex lens) and planar delay lines made of Plexiglas was *D_l_* = 17 mm.

An investigation of the aluminium sample with artificial defect along the azimuth cross-section (*y* axis) was performed using the linear scanning of ULPAT and two different configurations of specially manufactured delay lines: the first configuration with the beam focused in the elevation plane by the lens (convex) and the second without the focusing effect in the elevation plane.

**Figure 4:**
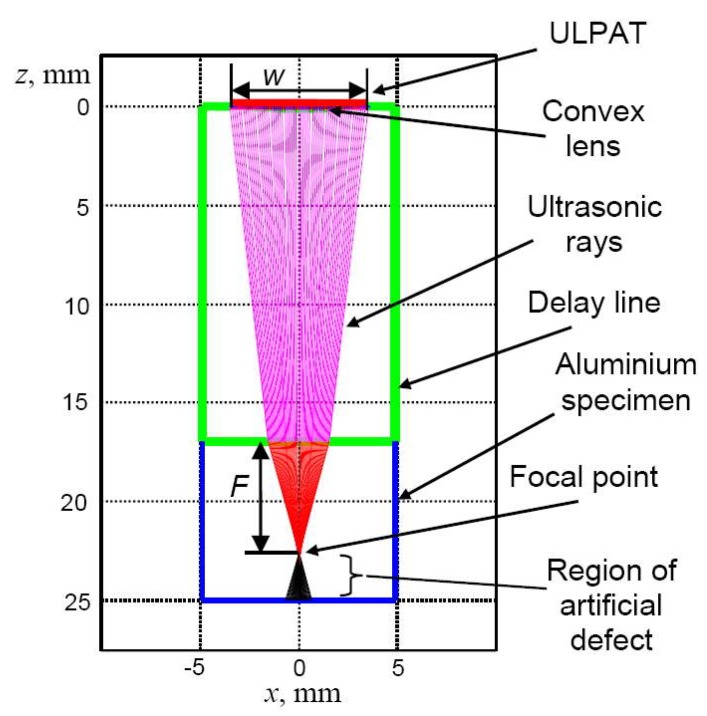
Estimation of the focal point *F* = 6 mm by the ultrasonic phased array transducer (ULPAT) with convex lens in the aluminium sample with a thickness of *D_sp_* = 8 mm.

The focus of the ULPAT in the elevation plane was simulated (analytically) using the ray tracing method (realized in MATLAB, R2013a) (Figure 4) [15]. Simulated results with the delay line thickness *D_l_* = 17 mm and the sample thickness *D_sp_* = 8 mm obtain the following: radius of lens *R* = 26 mm, focusing depth of *F* = 6 mm, the lateral dimension *W* = 7 mm of elevation (passive aperture) (Figure 4). The parameters used in the simulation were: *c*_1_ = 1470 m/s (water filling the cavity between ULPAT and delay line), *c*_2_ = 2680 m/s (delay line of Plexiglas), *c*_3_ = 6350 m/s (aluminium).

**Figure 5:**
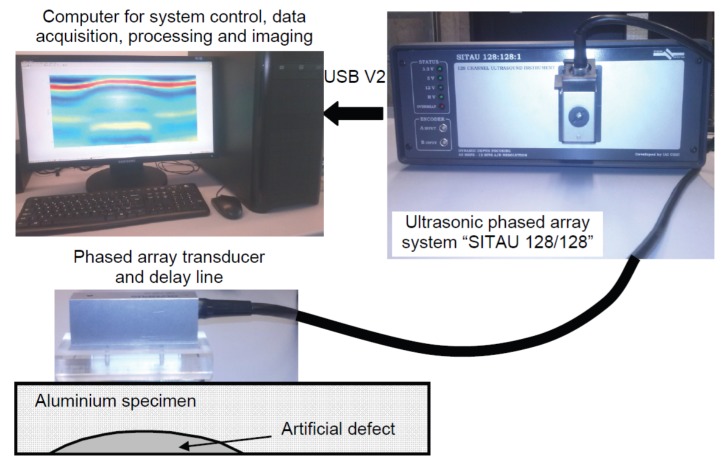
Images of the experimental setup.

**Figure 6:**
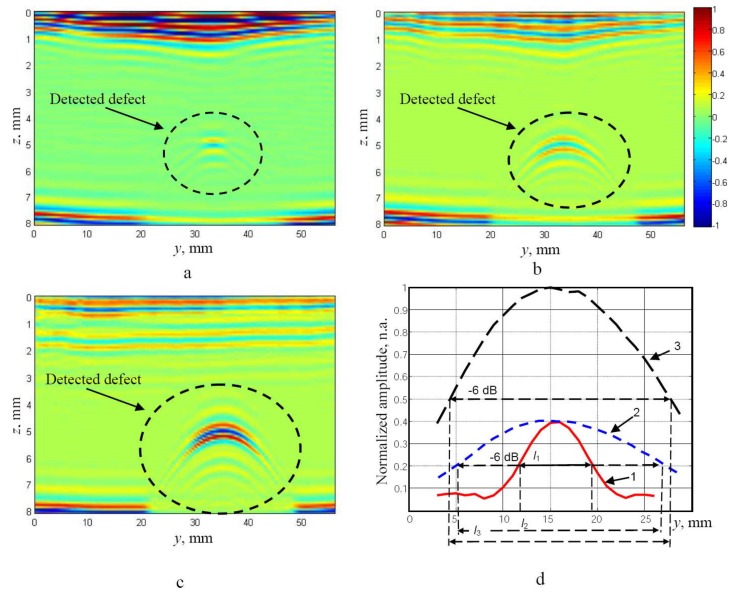
Experimentally obtained B-scan images: (**a**): in the case without focusing, (**b**): focusing the ULPAT in the active aperture (azimuth cross-section) only, (**c**): dual focused beam in the elevation and azimuth cross-sections. The peak amplitudes (normalized) of the reflections from the artificial defect (**d**): 1—without focussing, 2—with focusing in the azimuth cross-section, 3—with focusing in the elevation and azimuth cross-sections. Widths of the detected defect (marked by *l*_1_, *l*_2_ and *l*_3_) are estimated at the −6 dB level in respect of maximums of the normalized amplitudes for different configurations of beam focusing.

The experimental testing was performed using an Olympus ULPAT, type 10L128-64X7-l2-P-2.5-HY (Olympus, Waltham, MA, USA). The parameters of the technical specification of the ULPAT were an array with 128 elements, lateral dimensions (length) of the phased array active aperture *A* = 63.95 mm, the lateral dimension *W* = 7 mm of the elevation (passive aperture), the inter-element pitch of *p* = 0.5 mm, the width of the single transmitting–receiving element of the ULPAT *e* = 0.45 mm and the spatial gap between neighbouring transmitting–receiving elements *g* = 0.05 mm [15]. The excitation of the ULPAT possessing a central frequency of 10 MHz and acquisition of the reflected wave signals were performed by the multi-channel ultrasonic measurement system “Sitau 128/128″ (manufactured by Dasel Sistemas, Madrid, Spain) [15]. The active aperture of the ULPAT was scanned by electronic commutation in linear mode along the specimen with the spatial scanning step equal to the width of single transmitting–receiving element (0.5 mm) [15]. The pulse (duration 50 ns) for the excitation of each element of ULPAT within the active aperture was individually delayed by the particular time delay value of the calculated focal law [15]. The focusing of the ultrasonic beam excited by the ULPAT in the active aperture (the azimuth cross-section) of 16 elements was performed at the distance of 6 mm below the surface of the specimen under investigation. For acoustic coupling between the active (transmitting–receiving) surface of the ULPAT and the delay line (with convex lens and without), and also between the surface of the delay line and the specimen, the coupling gel was used. Images of the experimental setup are presented in Figure 5. The experimentally obtained results of the contact type linear scanning of the aluminium specimen, such as the peak amplitudes (normalized) and the B-scan images of the reflected ultrasonic waves signals from the artificial defect are presented in Figure 6a–d.

The B-scan images presented in Figure 6a–c were obtained in the case without focusing (Figure 6a), in the case of the electronic focusing of the ULPAT in the active aperture (azimuth cross-section) only (Figure 6b) and dual focusing in the elevation and azimuth cross-sections (Figure 6c). Figure 6d presents the peak amplitudes of the reflections from the internal artificial defect: 1—without focusing, 2—with focusing in the azimuth cross-section, 3—with a focused ultrasonic beam in the elevation and azimuth cross-sections.

The presented curves were normalized according to the maximal peak amplitude of the reflected wave signal from the artificial defect with dual focussed beam in the elevation and azimuth cross-sections (Figure 6d). The widths of the detected defect along axis *y* (marked by *l*_1_, *l*_2_ and *l*_3_) are estimated at the −6 dB level in respect of maximums of normalized amplitudes of reflections for different cases of focusing (Figure 6d). The obtained values of the detected defect width and relative errors were *l*_1_ = 7.5 mm (71.7%), *l*_2_ = 22.5 mm (15.1%) and *l*_3_ = 23.5 mm (11.3%). The lowest relative error (11.3%) of the detected defect width was observed in the case of the dual focused beam in the elevation and azimuth cross-sections.

CIVA 2020 (Simulation Software for Non-Destructive Testing (NDT)), which is developed by “Extende” (EXTENDE S.A., Massy, France) for the development, analysis and validation of NDT procedures of different objects [16,17], was used after the real experiment in order to perform a verification of the quantitative evaluation of the resolution enhancement and to compare with experimentally obtained values. The parameters of the ULPAT of 10 MHz frequency and 128 elements used in CIVA simulation are the same as described in the previous paragraph related to configuration of the convex lens and experimental investigation of the aluminium specimen. The focusing of ultrasonic beam excited by the ULPAT in active aperture (the azimuth cross-section) of 16 elements was performed at the distance of 6 mm below the surface of the specimen under investigation. The active aperture of the ULPAT was scanned by electronic commutation in linear mode with the spatial step 0.5 mm.

**Figure 7:**
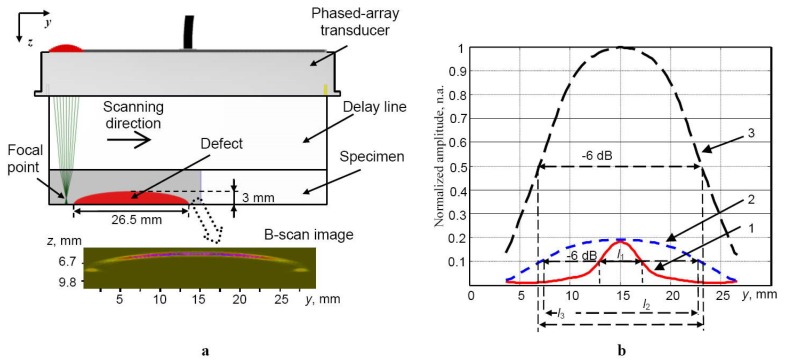
Simulations of the ultrasonic B-scan image obtained by the ULPAT with the delay line (material Plexiglas) within the specimen under investigation in the case of the linear electronic scanning along the axis *y* (azimuth cross-section) performed using CIVA (Simulation Software for Non-Destructive Testing) software (**a**) and the peak values of the reflections from the defect (**b**): 1—in the case without focusing, 2—focusing the ULPAT in the active aperture (azimuth cross-section) only, 3—dual focusing in the elevation and azimuth cross-sections.

The artificial defect was modelled as the real crescent-shaped defect in the aluminium sample (Figure 7a). The results of the simulation, such as the peak values of the reflections from the artificial defect and the B-scan images, are presented in Figure 7a,b. In Figure 7b, the presented curves were normalized according to the maximal peak amplitude of the signal reflected from the artificial defect with the dual focused beam in the elevation and azimuth cross-sections. Experimental and modelling results show that the resolution enhancement, higher amplitude of the reflections (2.5 times or +8 dB from the experiment, five times or +14 dB from the simulation) and spatial improvement in the defect detection are achieved with dual focusing of the ULPAT in the azimuth and elevation cross-sections (Figure 6d and Figure 7b, Curve 3). The widths of the detected defect along axis *y* (marked by *l*_1_, *l*_2_ and *l*_3_) are estimated at the −6 dB level in respect of maximums of the normalized amplitudes for different cases of focusing (Figure 7b).

The obtained values of the detected defect width and relative errors were *l*_1_ = 4.5 mm (83%), *l*_2_ = 15.05 mm (43.2%) and *l*_3_ = 16.2 mm (38.9%). The lowest relative error (38.9%) of the detected defect width was obtained in the case of the dual focused beam in the elevation and azimuth cross-sections as well. Therefore, the advantage of the dual focusing of ULPAT, in the case of detecting defects possessing spatially complex geometry and measuring their dimensions, was proved by corresponding experimental and simulation results. At the same time, it can be concluded that the proposed method has higher spatial resolution compared to standard beam focusing technique (in the azimuth cross-section only).

## 3. Experimental Inspection of the Multi-Layered GLARE Specimen

**Figure 8:**
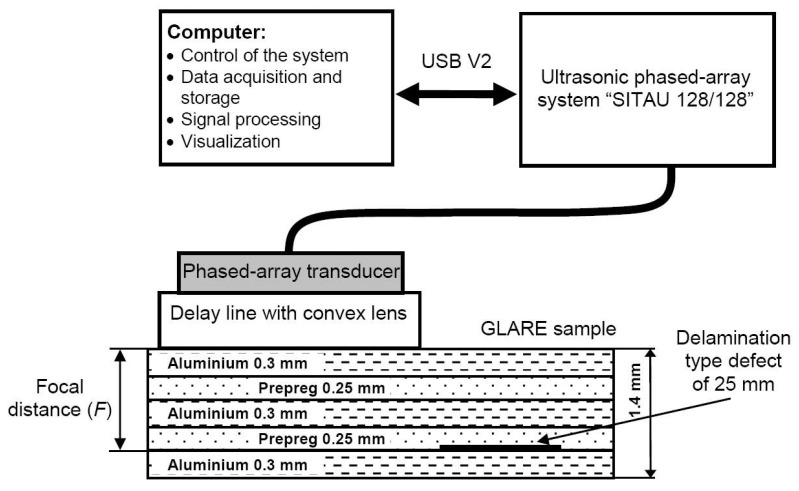
Experimental setup of the defects detection in the GLARE sample using the ULPAT and particular Plexiglas delay line in the following cases: focusing in the azimuth cross-section and with dual focused beam in the elevation and azimuth cross-sections using the convex cylindrical lens (the radius of the curvature is *R* = 17 mm).

For verification of the advantages of the proposed dual focusing method, the method was applied for the investigation of thin multi-layered composite (GLARE 3-3/2). The setup of experimental investigation of the defects detection in GLARE sample using the ULPAT and Plexiglas delay line is presented in Figure 8. Total thickness of the sample is *d* = 1.4 mm. The delamination type internal defect of composite panel was imitated as the artificial circularly shaped insert of Teflon between the fourth (prepreg) and fifth (aluminium) layers.

For laminates like GLARE, the most relevant defects that should be detected by the ultrasonic technique are porosity, voids and delamination [8,18,19,20,21]. Detection of delamination defects in such a multi-layered structure is problematic due to the thinness of these layers. The GLARE sample is constructed by stacking three thin (thickness 0.3 mm) aluminium alloy layers with 0°/90° glass fibre reinforced composite prepreg layers (thickness of 0°/90° prepreg combination is 0.25 mm) between the aluminium layers.

Due to the direct propagation and reflection of ultrasonic waves from the multi-layered structure possessing adjacent layers of different acoustic properties, the average value of the ultrasound velocity *c_av_* needs to be determined [13,21]. The analytical expression for calculation of average value of ultrasound velocity is obtained by [13,21]:

(6) $$c_{av}=\mathrm{MVF}\cdot c_{al}+(1-\mathrm{MVF})\cdot c_{pr}$$

where MVF is the Metal Volume Fraction, *c_al_* is the ultrasound velocity of longitudinal waves in aluminium layers (*c_al_* = 6320 m/s) and *c_pr_* is the ultrasound velocity of longitudinal waves in prepreg layers (*c_pr_* = 2700 m/s). The MVF is calculated as the ratio of summed up thicknesses of aluminium layers and the total thickness of the GLARE sample [13,21]:

(7) $$\mathrm{MVF}=\frac{\sum_{1}^{p}d_{al}}{d}$$

where *d_al_* is the thickness of each single aluminium layer (*d_al_* = 0.3 mm), *p* is the number of aluminium layers (*p* = 3) and *d* is the total thickness of the sample (*d* = 1.4 mm). The MVF for the analysed GLARE 3-3/2 sample is MVF = 0.643 and the average velocity *c_av_* = 5028 m/s.

There was a question related to the determination of the focusing of the ULPAT in two orthogonal cross-sections, because it was necessary to evaluate an average velocity of longitudinal ultrasonic waves in the multi-layered sample. If the focal point is adjusted to be on the bottom surface of the sample, the MVF and average ultrasound velocity *c_av_* should be the same as previously estimated. However, when focusing is adjusted at the selected focal distance *F* within the sample (e.g., at particular interface), MVF and average ultrasound velocity must be recalculated. The propagation distances of bulk ultrasonic waves in different mediums of adjacent layers up to the artificial delamination type defect and appropriate theoretical values of ultrasound velocities (e.g., in 2 aluminium and 2 prepreg layers) need to be taken into consideration. After recalculation, the values of MVF = 0.546 and average velocity *c_av_* = 4676 m/s are obtained.

On the other hand, such delamination type defects are always perpendicular to the direction of the longitudinal ultrasonic wave propagation, and particular reflections always occur. Therefore, it was important to apply the proposed method for detecting such internal defects. Moreover, the defects are distributed in the near-field of the ultrasonic transducers array and the thickness of the layers are close to the wavelength of the ultrasonic wave.

The experimental investigation was performed using the same ultrasonic multi-channel measurement system “Sitau 128/128″ and an Olympus ULPAT of 10 MHz (10L128-64X7-l2-P-2.5-HY). The focusing of the ULPAT in the azimuth cross-section was performed by using 16 elements of the active aperture at the distance of the focal point (*F* = 1.1 mm) below the front surface of the GLARE specimen (Figure 8). The active aperture of the ULPAT was scanned by electronic commutation in linear mode along the specimen with the spatial step of scanning equal to the width of a single transmitting–receiving element (0.5 mm). The average value of ultrasound velocity at the selected focal distance *F* was *c_av_* = 4676 m/s. The focusing of the ULPAT in the elevation cross-section was performed using the specially designed delay line with a convex cylindrical lens. The radius of the lens cylindrical curvature was *R* = 17 mm and *D_l_* = 20 mm.

The GLARE sample was scanned by linear scanning in different ways (Figure 8): 1—using a simple delay line and the ULPAT electronically focusing at focal point *F* and 2—using delay line with a convex lens and the ULPAT electronically focusing at focal point *F*.

**Figure 9:**
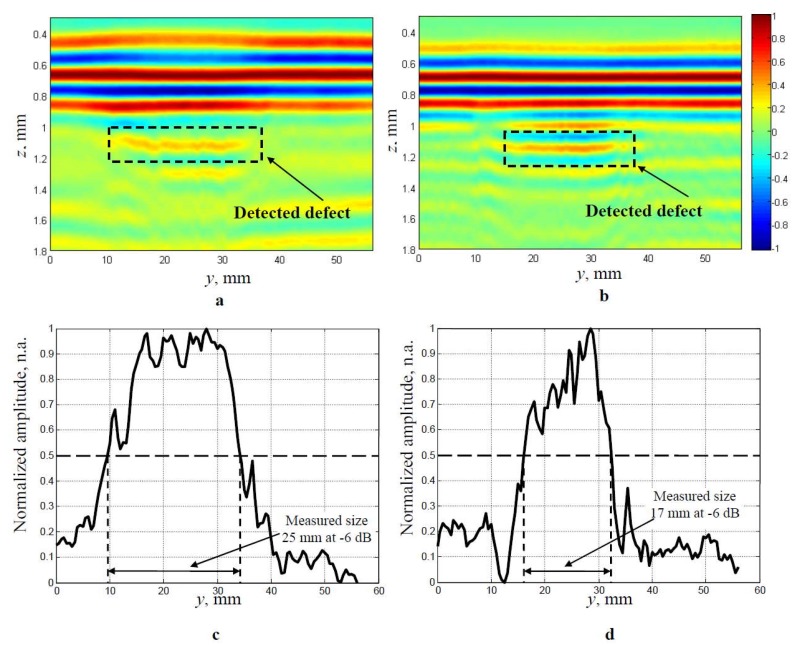
Experimentally obtained B-scan images (**a**,**b**) and the normalized peak amplitudes (**c**,**d**) of the delamination type defect of 25 mm detected in the multi-layered GLARE sample by a dual focused beam in the elevation and azimuth cross-sections (**a**,**c**) and focusing in the azimuth cross-section (without a lens) only (**b**,**d**).

The experimental results of detection of the artificial defect (diameter 25 mm) in the GLARE sample obtained using delay line with implemented convex cylindrical lens, the simple delay line (without a lens) and the ULPAT electronically focusing at focal point *F* = 1.1 mm, are presented in Figure 9a–d. The B-scan image and the peak amplitudes of the reflected ultrasonic signals from the defect, obtained using the delay line with a convex cylindrical lens and the ULPAT electronically focusing at focal point *F* = 1.1 mm are presented in Figure 9a,c. The B-scan image and the peak values of the reflections from the defect obtained using simple delay line (without lens) and the ULPAT electronically focusing only at the focal point *F* = 1.1 mm are presented in Figure 9b,d.

In the case of the dual focusing in the elevation and azimuth cross-sections, the defect is detected with a 25 mm diameter (at −6 dB level) and corresponds well to the actual dimensions in Figure 9c. However, in the case of the focusing in the azimuth cross-section only, the delamination defect is detected with a 17 mm diameter (at the −6 dB level) and the relative error of diameter estimation is 32% (Figure 9d).

## 4. Discussion and Conclusions

A solution based on the application of the specially designed delay line containing a fluid-based convex cylindrical lens [15] was proposed to achieve the spatial dual focusing effect, enhance the 3D spatial resolution of ULPAT and increase the accuracy of ultrasonic inspection and sizing of internal defects of thin multi-layered composite components of the aircraft fuselage.

The lens enables us to achieve the spatially focussed ultrasonic beam in the elevation cross-section at a particular distance, while in the azimuth cross-section, the excited ultrasonic beam is electronically focused on internal defects and scans to obtain the B-scan image [15]. The proposed focusing technique of the ULPAT (10 MHz, 128 elements) in the elevation cross-section was demonstrated using the geometrical ray tracing method, while the focused ultrasonic beam at a defined range inside the specially prepared aluminium specimen possessing internal defects of spatially complicated geometry (crescent-shaped) was investigated using a CIVA simulation package. Widths of the detected defect were estimated at the −6 dB level in respect of maximums of normalized amplitudes for different cases of focusing, such as without focusing, focusing in the azimuth cross-section only and proposed dual focusing in the azimuth and elevation cross-sections. The lowest relative error (e.g., 11.3%) of the detected defect width was observed in the case of dual spatial focusing of ULPAT in the elevation and azimuth cross-sections. The simulation results and experiments demonstrate the resolution enhancement, higher amplitude of the reflections (e.g., by 2.5 times or +8 dB) and spatial improvement in the defect detection even in the case of the non-perpendicular incidence of ultrasonic waves to the complex geometry surface of the internal defect.

The experimental verification of the proposed technique for spatial focusing was performed by the application of testing of GFRP-metal based multi-layered composite sample GLARE 3-3/2. The sample consists of an artificial delamination type defect by appropriate Teflon insert, possessing a diameter of 25 mm. Compared to the limitations of conventional ULPAT, the relative error 32% (at −6 dB level) of the lateral defect dimensions estimation was completely reduced. Therefore, it can be stated that the proposed solution and inspection technique based on a special delay line is suitable to be applied for contact-type testing of multi-layered composite specimens made of dissimilar materials possessing different material properties (e.g., lightweight metals and GFRP). Furthermore, it is suitable for the in-service inspection of the assembled aircraft fuselage components (e.g., based on GLARE, etc.) with the increased spatial resolution, higher accuracy of defect sizing and the possibility to be applied in the case of single-side access only.

On the other hand, there are some limitations with the application of the proposed method in the case of detection of non-homogeneities for other types of composite objects. The first limitation is based on the selection of excitation frequency for ultrasonic phased array transducers (ULPAT). In the presented case, the optimal frequency for inspection of the GLARE structure was selected 10 MHz [13,14]. For other types of multi-layered composite structures, possessing different thicknesses, this frequency value may be lower or higher.

The second limitation is based on focal point estimation in the elevation and azimuth cross-sections within the sample under investigation. However, in the case the investigated multi-layered composite structure is thin (total thickness of the sample is close to a few wavelengths of ultrasonic wave), the obtained results indicate that spatial dual focusing adjusted on the bottom surface or on particular interfaces of the sample is sufficient to detect defects at various depths. The average ultrasound velocity in the investigated sample is calculated according to the analytical expression (Equation (6)).

The third limitation—internal delamination defects in multi-layered structures may be localised at any internal interface between the adjacent thin layers and the overlapping of the reflections may occur. To solve this limitation, in the previous work we proposed the signal processing method based on the developed adaptive numerical model of ultrasonic waves propagation through a multi-layered structure and selection of particular segments of the signal in frequency and time domains [22]. The results presented prove that the proposed approach enables us to achieve the improved resolution of internal delamination-type defects of multi-layered structures [22]. A similar model was applied to characterize porosity inside GLARE samples and retrieve information about the thickness of the layers as well [14,19].

The proposed technique of increased spatial resolution of ULPAT and application examples are important in the field of ultrasonic material characterization in order to solve the actual tasks that always arise during the different stages of the lifecycle of composites and joined dissimilar materials. It covers the demand for characterization and quality control in the designed and manufactured unique prototypes of composites in a research lab, and the characterization after tensile or fatigue tests and quality control at particularly defined manufacturing steps during the mass production in a factory. In addition, the NDT procedures to prevent faults, accidents and safety assurance of already in-service exploited composite-based lightweight constructions (e.g., components of the aircraft fuselage) could be improved.

The advantages of the technique in the case of contact type application and single-side access to the surface of thin multi-layered composites:

- Non-invasive evaluation of interface (bonding) quality between the adjacent layers (e.g., joined dissimilar materials: aluminium and prepreg);
- Detection of internal non-homogeneities within the structure of the multi-layered specimen (e.g., quality control and characterization of intermediate prepreg layer);
- For the already assembled components detection of the thickness variations of the individual layers (e.g., aluminium or prepreg);
- For the already assembled components simultaneously detection of presence of the internal defects (e.g., porosity, delamination or disbond), estimation their depth within the specimen (e.g., particular layer or interface) and the size or lateral dimensions of the defect as well.

Considering all the discussed results we obtained, potential advantages, taking into account the limitations and the practical benefits of the proposed solution, it can be stated that it does not require quite expensive and complex ultrasonic NDT or measurement systems. In addition, the solution we proposed is universal and does not directly depend on specifics of multi-channel ultrasonic hardware (measurement system). It could be implemented using any phased array flaw detector and linear phased array transducer (e.g., central frequency of 10 MHz for GLARE specimen) combined with manufactured special delay line for contact applications (possessing a liquid-filled convex lens). Fabrication parameters of the delay line depend on the dimensions of appropriate phased array transducers (active surface for emission–reception), specificity of the multi-layered composite object under investigation (e.g., ultrasound velocity of bulk ultrasonic waves in each layer and number of layers) and required depth of focusing inside the object. The radius of convex cylindrical lens and appropriate thickness of the delay line are necessary to be calculated using the derived expressions and the ray tracing approach presented in the article. This marks a further step towards practical implementation, covering the manufacturing procedure (e.g., using CNC machine-tool) of the special delay line.
